# *Mycobacterium tuberculosis* Modulates the Gene Interactions to Activate the HIV Replication and Faster Disease Progression in a Co-Infected Host

**DOI:** 10.1371/journal.pone.0106815

**Published:** 2014-09-08

**Authors:** Jaideep S. Toor, Sukhvinder Singh, Aman Sharma, Sunil K. Arora

**Affiliations:** 1 Department of Immunopathology, Postgraduate Institute of Medical Education & Research, Chandigarh, India; 2 Department of Internal Medicine, Postgraduate Institute of Medical Education & Research, Chandigarh, India; Tulane University, United States of America

## Abstract

Understanding of the chronic immune activation, breakdown of immune defense and synergistic effect between HIV and *Mycobacterium tuberculosis* (*Mtb*) may provide essential information regarding key factors involved in the pathogenesis of HIV disease. In this study, we aimed to highlight a few of the immunological events that may influence and accelerate the progression of HIV disease in the presence of co-infecting *Mtb*. A cross-sectional study was performed on cohorts, including anti-tubercular therapy (ATT) naïve active pulmonary tuberculosis (PTB) patients, antiretroviral therapy (ART) naïve HIV-1 infected individuals at different stages of disease, ATT and ART naïve HIV-PTB co-infected individuals and healthy controls. A significantly higher T-regulatory cell (Treg) frequency coupled with the high FoxP3 expression in the CD4 T-cells indicated an immunosuppressive environment in the advance stage of HIV-1 infection. This is further substantiated by high HO-1 expression favoring TB co-infection. Functionally, this change in Treg frequency in HIV-1 infected individuals correlated well with suppression of T-cell proliferation. *Mtb* infection seems to facilitate the expansion of the Treg pool along with increased expression of FoxP3, specifically the variant-1, as evident from the data in HIV-1 co-infected as well as in patients with only PTB. A significantly lower expression of HO-1 in co-infected individuals compared to patients with only HIV-infection having comparable CD4 count correlated well with increased expression of CCR5 and CxCR4 as well as NF-κB and inflammatory cytokines IL-6 and TNF-α, which collectively may contribute to enhanced viral replication and increased cell death, hence faster disease progression in co-infected individuals.

## Introduction

*Mycobacterium tuberculosis (Mtb)* continues to be one of the most dreaded pathogens around the world and its co-habitation with HIV in a common host fuels both the infections. Of the estimated 39 million people living with HIV, about one-third are estimated to have concomitant latent tuberculosis (Global Tuberculosis Control, WHO, 2010). Depending on the prevalence of HIV in a population, the risk for active tuberculosis becomes 20–37 times higher in individuals living with HIV than in the general population. India has approximately 2.1 million cases of HIV/AIDS predominantly infected with HIV-1 subtype-C [1].

The immune response towards any pathogen is regulated not to cause excessive damage to the host while leading to containment of infectious agent. During HIV-1 infection, this precise balance of the host immune response and its regulation gets disturbed, making the environment favorable for other opportunistic pathogens, especially *Mtb*. Regulatory T-cells (Tregs) are an important cell population that maintains central tolerance as well as controls the overall response to the invading pathogens, including viruses, however, their role during HIV infection remains controversial. The natural immune-suppressive ability of Tregs may be playing dual role during HIV infection, being harmful to the host for suppressing the viral- specific immune response [2], [3], [4], [5] on one hand and being beneficial at the same time for suppressing the hyperimmune activation state characteristic of this disease [6], [7], [8], [9]. In order to ascertain their behavior in a co-infected host and also if *Mtb* has any modulatory effect on Tregs that may change the course of HIV infection in the co-infected host, we have investigate the role of Tregs during HIV infection alone or during *Mtb* co-infection. Overall, we have address a few of the issues related to deadly synergism between HIV-1 and *Mtb* by focusing on the sensitive balance between immune-suppressive genes like HO-1 and FoxP3 as well as immune-stimulatory genes like NF-κB and how this balance affects the expression of HIV-1 co-receptors, CCR5 and CxCR4 on CD4 T-cell subpopulations. Our findings highlight a pathway in which *Mtb* down regulate the expression of HO-1 leading to up-regulation of the redox sensitive NF-κB that in turn induces the replication of HIV-1 proviral genome in PTB co-infected individuals, besides increasing the expression of HIV co-receptors CCR5 and CxCR4.

## Material and Methods

### Ethics statement

The study was approved by the Institutional Ethics Committee (IEC) of PGIMER, Chandigarh, India and 10 ml peripheral blood was obtained from each enrolled subject after an informed written consent. There were no minor/children recruited for this study.

### Study subjects

The study was conducted on cohorts, including 27 HIV-1 infected patients, 12 pulmonary tuberculosis patients (PTB), 8 HIV-PTB co-infected patients (HIV-PTB), and 20 healthy controls (HC). HIV-1 infected patients, confirmed positive by three serologic tests as per National AIDS Control Organization (NACO, Govt. of India) guidelines, were enrolled from the Integrated Counselling and Testing Centre (ICTC) in the Department of Immunopathology, PGIMER Chandigarh, India. At the time of recruitment, the patients were interviewed by a counsellor to obtain informed consent and ascertain therapy naïve status. The status of the disease was assessed by absolute CD4 cell count monitored by flow cytometry using BD Tritest containing antibody conjugates CD3-FITC/CD4-PE/CD45-PerCP with BD Trucount tubes (BD Biosciences, San Jose, USA). PTB and HIV-PTB patients, confirmed positive for *Mtb* infection by chest x-ray and sputum smear positivity were recruited from DOTS (Directly observed therapy-short course) centre at our hospital. The peripheral blood mononuclear cells (PBMCs) were isolated from the heparinized blood by Ficoll-Hypaque density gradient centrifugation (HiMedia, Mumbai, India).

### Treg Immunophenotyping

**Figure 1:**
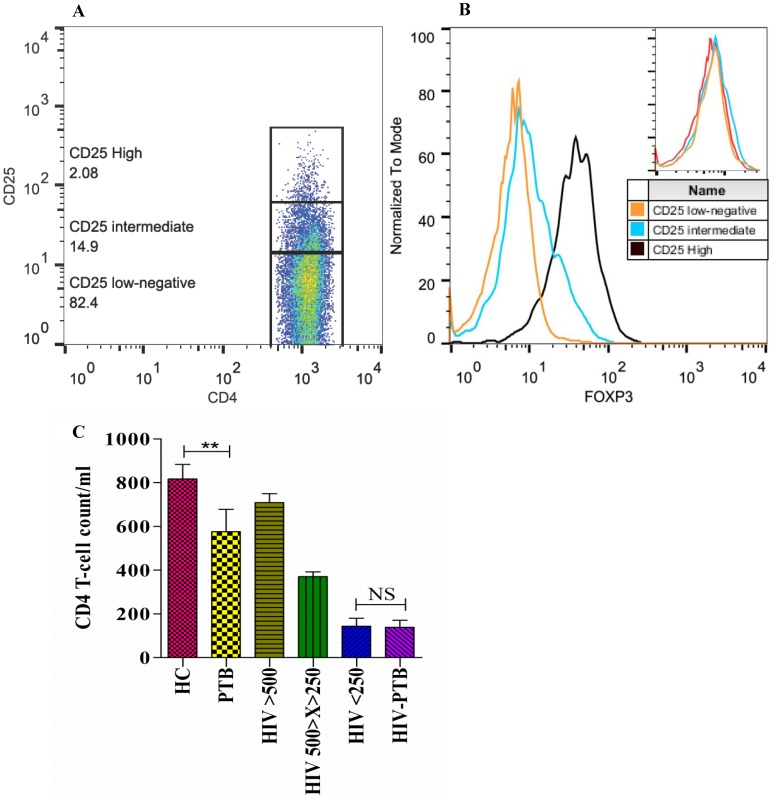
Lower CD4 T-cell count in PTB individuals as compared to healthy controls. **A.** Representative dot plot showing gating strategy for immunophenotyping of the three subpopulations of CD4^+^ T-cells based on CD25 expression as: CD25^high^, CD25^intermediate^ and CD25^low/negative^. **B**. Representative overlay histogram showing intracellular FoxP3 expression in different CD4^+^ T-cell subpopulations with isotype control (upper right). **C**. Bar diagram showing mean±SEM of CD4 counts of each studied group. HC: Healthy control; PTB: pulmonary tuberculosis; HIV >500: HIV-1 infected individuals with CD4 count above 500 cells/µl; HIV 500>X>250: HIV-1 infected individuals with CD4 count between 500 and 250 cells/µl; HIV <250: HIV-1 infected individuals with CD4 count less than 250 cells/µl. Significant differences are represented as the p value <0.01 (**), NS: (non-significant).

Freshly isolated PBMCs were immunophenotyped for Treg number, FoxP3 expression (MFI) and CCR5/CxCR4 expression using fluorochrome-conjugated monoclonal antibodies (mAb): anti-CD4 PE/FITC, anti-CD25 PE-Cy7, anti-CCR5 PE, and anti-CxCR4 PE (BD Bioscience, San Jose, CA) for cell-surface markers in combination with intracellular Fork-head box protein 3 (FoxP3) mAb conjugated with Alexa488 (eBioscience, San Diego, CA). Samples were acquired into a flow cytometer (FACS Calibur, BD, USA) and analyzed using Cell-Quest (BD Bioscience)/FlowJo (Treestar) softwares. For further analysis, patients were categorized on the basis of level of surface CD25 expression on CD4^+^ T-cells viz: CD4^+^CD25^high^ (top 2% with high CD25 expression), CD4^+^CD25^intermediate^ (middle 15% with intermediate CD25 expression) and CD4^+^CD25^low/negative^ (lower 83% with very low or no expression of CD25) cells (**Figure 1A & 1B**). Absolute counts for different populations were calculated from the total lymphocyte count in the whole blood.

### T-cell proliferation assay

To examine the functional defects of T-cells in these cohorts, proliferative capability of T cells was assessed on stimulation with mitogen-phytoheamagglutinin [PHA (10 µg/ml] by determining the extent of ^3^H-thymidine incorporation. Briefly, 1×10^5^ PBMCs/well were plated into 96-well plates in triplicates in complete medium [RPMI 1640 (Sigma) medium supplemented with 10% heat-inactivated FCS, 20 IU/ml penicillin, 20 µg/ml streptomycin, 25 mM HEPES, and 2 mM L-glutamine] incubated for 96 hours at 37°C in 5% CO_2_ and humidified atmosphere, either in presence or absence of PHA as the stimulant. Eighteen hours prior to termination of the culture, 1 µCi of ^3^H-thymidine (BARC, India) was added to each well. The cells were then harvested on glass fibre mats (Skatron, Belgium) and radioactivity was counted in a liquid scintillation β-counter (Beckman, USA). All measurements were conducted in triplicate and the results were expressed as the stimulation index (SI), which represented the mean cpm (counts per minute) in the presence of mitogen divided by mean cpm in the absence of the mitogen.

### Detection of cytokine in culture supernatants

Patient plasma samples and supernatants were collected from the PHA stimulated PBMC cultures (as above) before addition of ^3^H-thymidine for estimation of cytokines. The cytokines TNF-α and IL-6 were measured using Th1/Th2 Cytokine CBA Kit II (BD Cytokine Bead Array).

### Analysis of Mitochondrial Membrane Potential (Δψ_m_)

Cell death was evaluated flowcytometrically by measuring mitochondrial membrane potential (Δψm) with J-aggregate forming lipophilic action 5, 5′, 6, 6′-tetraethylbenzimidazolocarbocyanine iodide (JC-1) dye (Sigma-Aldrich, USA). Briefly, 2 ml whole blood was lysed with RBC lysing buffer (Himedia, Mumbai, India) and isolated leukocytes were washed with phosphate buffer saline (PBS, Himedia, Mumbai, India) [10]. Finally, 1×10^6^ cells/ml were resuspended in PBS before staining with JC-1 dye (2.5 µM) and incubated at 37°C (5% CO_2_ humidified atmosphere) for 15 min in dark. Stained cells washed and resuspended, were acquired immediately on flow cytometer (FACSCanto II) and the data was analyzed using software FACSDiva Version 6.1.3 (BD Biosciences, USA). Lymphocytes were gated on forward scatter and side scatter to exclude debris and non-lymphoid cells. JC-1 fluorescence was analyzed on FL1 and FL2 channels for the detection of dye monomers (Green) and J-aggregates (Red), respectively. The dead cell gate was set up by the mitochondrial uncoupling agent protonophore carbonyl cyanide p-(trifluoromethoxy) phenylhydrazone (FCCP) (Sigma-Aldrich, USA). The treatment with the protonophore FCCP resulted in maximum decrease of the JC-1 fluorescence ratio and served as a positive control for disruption of mitochondrial membrane potential. The ratio of red/green fluorescence reflected mitochondrial trans-membrane potential. Δψm was expressed as median percent fluorescence intensity (FL2: FL1/FL2 FCCP: FL1 FCCP) ×100.

### Semi-quantitative estimation of gene expression by RT-PCR analysis

Total RNA was extracted from EDTA blood using the QIAmp RNA Blood Mini Kit (Qiagen, Germany). RNA was converted into cDNA using Revert Aid First Strand cDNA Synthesis Kit (MBI Fermentas, Lithuania). The first strand cDNA was used in PCR reaction for measuring the relative expression of various genes normalized against the expression levels of a house keeping gene GAPDH. The primers for FoxP3 splice variant 1 (|NM_014009.3, representing the longer transcript), splice variant 2 (NM_001114377.1; lacking an in-frame segment of the coding region), heme oxygenase 1 (NM_002133.1), CxCR4 (NM_001008540.1) and NF-κB (NM_003998.3) were as follows:

GAPDH forward: 5′-CAAGGTCATCCATGACAACTTTG-3′ and reverse: 5′-GTCCACCACCCTGTTGCTGTAG-3′; FoxP3 forward: 5'-CGGACCATGTTCTGGATGAG-3' and reverse: 5'-TTGTCGGATGATGCCACAG-3'; HO-1 forward: 5'-AGGCCAAGACTGCGTTCCT-3' and reverse: 5'-GCAGAATCTTGCACTTTGTTG -3'; CxCR4 forward: 5′- CTTCTACCCCAATGACTTGTGG-3′ and reverse: 5′- AATGTAGTAAGGCAGCCAACAG-3′; NF-κB forward: 5′-TTCACCAAGCCTGCCCTTGGAC-3′ and reverse: 5′-CTGTCTTGTGGACAACGCAGTGGAATTTTAGG-3′.

The thermal cycler was programmed as: denaturation at 95°C for 5 min, followed by 35 cycles at 95°C for 30 Sec and annealing at 58°C for GAPDH, 64°C for CxCR4, 60°C for FoxP3, 58°C for HO-1 and 62°C for NF-κB for 30 Sec, extension in all was carried at 72°C for 1 min, with a final extension step of 10 min.

### Statistical analysis

Data were expressed as mean ± standard error of mean (SEM) and ranges. The difference between groups was tested by one-way student's *t*-test. Correlation analysis was done using the non-parametric Spearman's rank correlation coefficient. Linear Regression Analysis was carried out and the residuals were calculated. The level of significance was set at p<0.05.

## Results

### Demographic and Immunological characteristics of study subjects

In this cross-sectional study we recruited a total of 67 subjects including 27 patients infected with HIV-1 only (age range: 21–53 years and CD4^+^ T-cell count range: 32–854 cells/µl), 12 patients with active PTB (age range: 21–65 years and CD4^+^ T-cell count range: 326–1368 cells/µl), 8 HIV patients having pulmonary tuberculosis also (HIV-PTB co-infected patients) (age range: 17–34 years and CD4^+^ T-cell count range: 30–273 cells/µl), and 20 healthy controls (age range: 22–40 years and CD4^+^ T-cell count range: 444–1375 cells/µl).

There was no significant difference between the mean CD4 count of HIV-1 infected individuals in the advanced stage of disease (CD4 count <250 cells/µl) and HIV-PTB co-infected individuals [CD4 count (mean±SEM cells/µl): 144±36 and 138±32 respectively] (**Figure 1C**). With similar immunological status in terms of CD4 count between the two above mentioned groups, comparisons were made to determine how PTB influences HIV-1 disease in co-infected individuals. The mean CD4 count in individuals with active pulmonary tuberculosis only (HIV-1/2 negative) was significantly lower than healthy controls [CD4 count (mean±SEM cells/µl): 577±102 vs 817±66, p = 0.0064], though well within the normal range.

### Increased Treg frequency coincided with poor T-cell proliferation in advanced stage of HIV disease with *Mtb* co-infection

**Figure 2:**
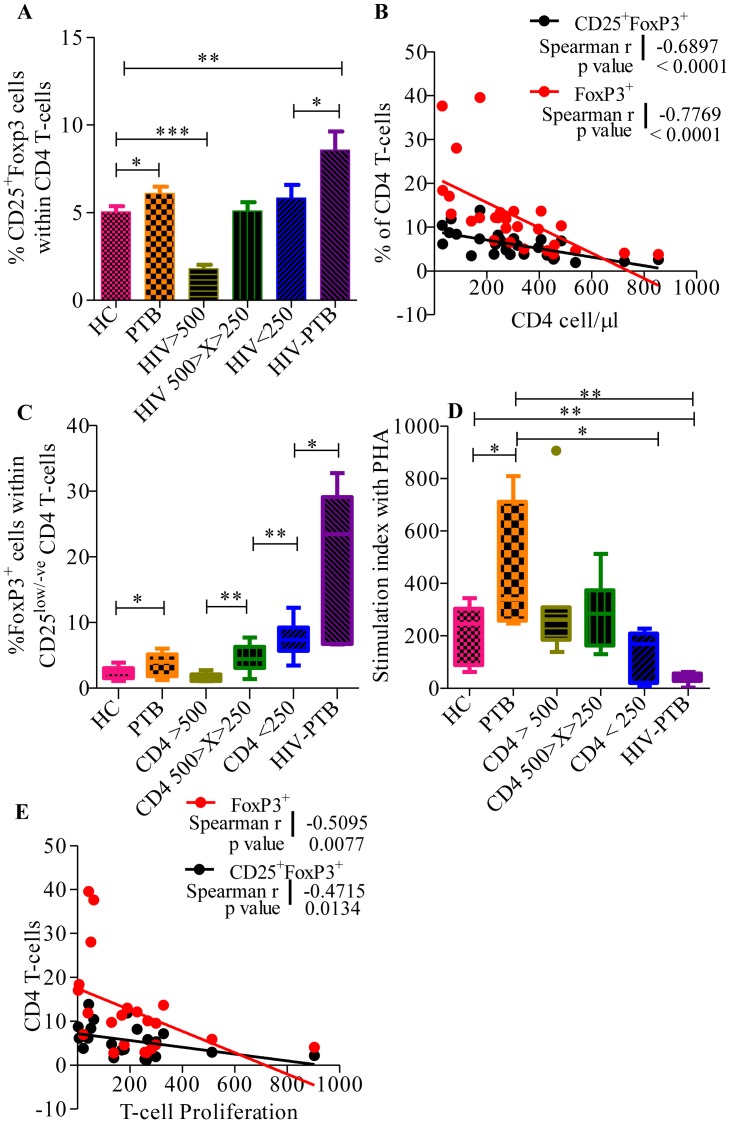
Higher Tregs frequency linked with PTB in individuals co-infected with or without HIV-1. **A.** Cumulative data depicting frequency of CD25^+^Foxp3^+^ Tregs within CD4^+^ T-cell in healthy controls (HC), patients with Pulmonary tuberculosis (PTB) and HIV-1 infected individuals with or without PTB co-infection. **B.** *Association of increased Foxp3^+^ CD4 T-cell frequency with HIV-1 disease progression (decrease in CD4 count)*. Dot plot showing significant negative correlation between T-cell proliferation and CD4^+^Foxp3^+^ (red dots, Spearman r = −0.7769, p value <0.0001)/CD25^+^Foxp3^+^ (black dots, Spearman r = −0.6897, p value <0.0001) frequency in HIV-1 infected individuals. The solid line represents the linear regression of points and dotted lines represents 95% confidence band for their mean values. Rho and p values are from the Spearman rank correlation test. **C.** *Increase in the FoxP3^+^CD25^low/-ve^ CD4 T-cells in PTB and HIV-PTB co-infected individuals*. Whisker box plot showing frequency of FoxP3 positive cells within CD25^low/-ve^ cells in the PBMCs of study subject groups. The horizontal line within the box indicates the median, the boundaries of the box indicate the 25^th^- and 75^th^ -percentile, and the whiskers indicate the highest and lowest values of the frequency of FoxP3 positive cells. **D.** *Low T-cell proliferation in HIV-PTB co-infected individuals*. Whisker box plot showing the proliferative T-cell response profile to PHA [stimulation for 96 hours] in PBMCs from healthy controls, patients with PTB and HIV-1 infected individuals with or without *M. tuberculosis* co-infection. Stimulation indices were determined by comparison with paired unstimulated cells from each individual. The horizontal line within the box indicates the median, the boundaries of the box indicate the 25^th^- and 75^th^ -percentile, and the whiskers indicate the highest and lowest values of the stimulation indices. **E.** *Association of increased Foxp3^+^ CD4 T-cells frequency with decreased T-cell proliferative response*. Dot plot showing significant negative correlation between T-cell proliferation and Foxp3^+^ (red dots, Spearman r = −0.5095, p value <0.0077)/CD25^+^Foxp3^+^ (black dots, Spearman r = −0.4715, p value  = 0.0134) in HIV-1 infected individuals. The solid line represents the linear regression of points and dotted lines represents 95% confidence band for their mean values. Rho and p values are from the Spearman rank correlation test.

There was a significantly higher frequency of CD25^+^FoxP3^+^ CD4 T-cells in PTB infected individuals with (mean±SEM: 8.57±1.07, p = 0.0012) and without (mean±SEM: 6.09±0.39, p = 0.035) HIV-1 co-infection when compared to healthy controls (mean±SEM: 5.04±0.33) (**Figure 2A**). Similar results were observed with significantly higher frequency of FoxP3 expressing CD4 T-cells in HIV-1 infected individuals with CD4 count <250 cells/µl (mean±SEM: 12.48±1.27, p = 0.001), HIV-PTB co-infected individuals (mean±SEM: 24.25±4.36, p<0.0001) and PTB individuals (mean±SEM: 9.45±0.74, p = 0.0146), when compared with healthy controls (mean±SEM: 7.29±0.38). The relationship between HIV-1 disease progression (in terms of CD4 count) with FoxP3+ CD4 T-cells and CD25+FoxP3+ CD4 T-cell was evaluated. Highly significant association was observed in HIV-1 infected individuals with increased FoxP3^+^ cell frequency as the disease progressed (Spearman r = -0.7769, p value <0.0001) (**Figure 2B**). This was partially contributed by a significant increase in the CD4^+^CD25^low/negative^ FoxP3^+^ cells (**Figure 2C**). This trend was also visible in the HIV-PTB co-infected individuals (median = 23.42, mean±SEM: 19.52±4.34, p = 0.049) when compared to only HIV infected individuals with similar CD4 counts (median = 7.37, mean±SEM: 7.63±1.05) (**Figure 2C**).

This change in the Tregs frequency correlated well with a significant decrease in the T-cell proliferation in response to PHA stimulation in HIV-PTB co-infected individuals (median = 43.1, mean±SEM: 39.90±10.00, p = 0.0011) when compared to healthy controls (median = 245.7, mean±SEM: 209.7±30.76) (**Figure 2D**). Within the HIV-1 infected group, T-cells from HIV-1 infected individuals in the early stage of disease (CD4 count >500 cells/µl) showed higher T-cell proliferation (mean±SEM: 332.9±97.74, p = 0.1596) as compared to healthy controls or HIV-PTB co-infected subjects, correlating well with a significantly lower frequency of Tregs in these patients (mean±SEM: 3.49±0.31, p = 0.0003). Overall, we observed that the T-cell proliferation was negatively correlated with frequency of CD25^+^FoxP3^+^ (Spearman r = −0.4715, p value  = 0.0134) and only FoxP3^+^ (Spearman r = −0.5095, p value <0.0077) CD4 T-cells in HIV infected individuals (**Figure 2E**). The above results indicate higher CD25^low/-ve^FoxP3^+^ cells and intact suppressive function of expanded Tregs in HIV-PTB co-infected patients.(instead we need a fig showing T-cell proliferation data in different gps)

### Higher intracellular FoxP3 protein levels in Tregs from PTB-HIV co-infected individuals is associated with higher FoxP3 variant 1 mRNA levels in PBMCs

**Figure 3:**
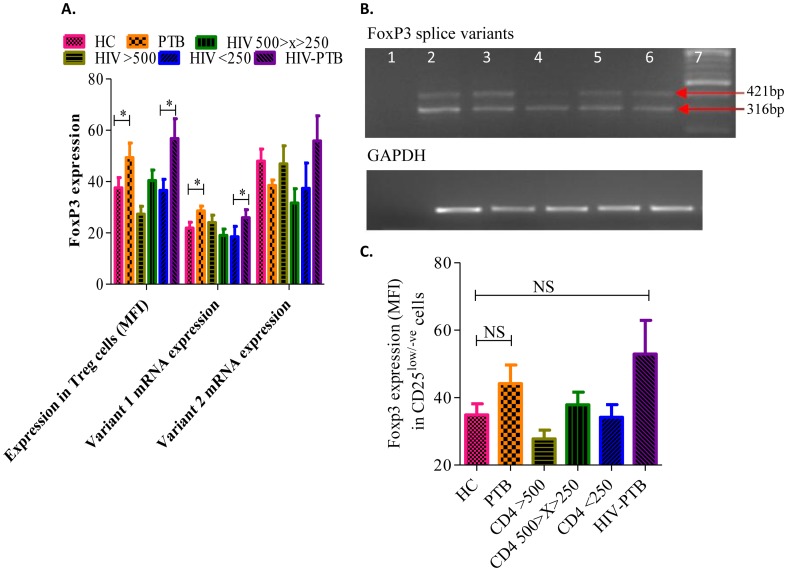
High FoxP3 expression in PTB patients with or without HIV-1 co-infection correlated with FoxP3 variant-1 mRNA expression only. **A**. Bar diagram depicting expression of intracellular Foxp3 within CD4^+^ T-cells, the relative expression of Foxp3 variant-1 and variant 2 mRNA (normalized using GAPDH levels) in all studied groups. Data were expressed as Mean±SEM and p-value < 0.05 (*) was considered significant. **B**. Representative 2% agarose gel image of RT-PCR products, showing mRNA levels of FoxP3 splice variant 1 (421 bp) & variant 2 (316 bp) and their corresponding housekeeping gene, GAPDH (180 bp) in PBMCs of HIV-1 infected individuals. Lane 1: Negative control, lanes 2-6: HIV-1 infected individuals and lane 7: 100 bp DNA ladder. C. *Higher FoxP3 expression in CD25^low/-ve^ cells from PTB patients with or without HIV-1 co-infection*. Whisker box plot showing levels of FoxP3 expression (MFI) in CD25^low/-ve^ cells in the PBMCs of study subject groups. The horizontal line within the box indicates the median, the boundaries of the box indicate the 25^th^- and 75^th^ -percentile, and the whiskers indicate the highest and lowest values of the FoxP3 expression.

Higher FoxP3 expression has been related to stability of Tregs and their intact suppressive functions. The FoxP3 expression was significantly higher in CD4^+^ Tregs from PTB patients (mean±SEM: 49.35±5.71, p = 0.0347) and HIV-PTB co-infected individuals (mean±SEM: 56.76±7.75, p = 0.0180) as compared to healthy controls (mean±SEM: 37.48±4.01) (**Figure 3A**). Interestingly, there was significantly higher Foxp3 protein level in Tregs from HIV-PTB co-infected individuals (mean±SEM: 56.76±7.75) in comparison to individuals infected with only HIV having comparable CD4 count of <250 cells/µl (mean±SEM: 36.60±4.30, p = 0.0361) indicating a possible role of *Mtb* in inducing Foxp3 expression. This significant difference correlates with only FoxP3 variant 1 mRNA expression as evident from its significantly higher expression in PTB (mean±SEM: 28.58±1.85, p = 0.0294) and HIV-PTB co-infected individuals (mean±SEM: 25.92±3.13, p = 0.0476) when compared to healthy controls (mean±SEM: 21.92±2.24) and HIV-1 individuals with similar CD4 count [<250 cells/µl (mean±SEM: 18.51±4.06)]. Though the overall expression level of FoxP3 variant-2 was higher than variant-1, we did not observe any significant difference in the expression levels of variant-2 among any of the studied groups (**Figure 3A & 3B**).

Significant increase in the frequency of CD4^+^CD25^low/negative^ FoxP3^+^ cells in PTB individuals with and without HIV-1 co-infection had higher FoxP3 expression in these cells (HIV-PTB median = 52.82, mean±SEM: 52.92±9.98 and PTB median = 42.83, mean±SEM: 44.13±5.52) when compared to healthy controls (median = 28.45, mean±SEM: 34.84±3.33) though statistically non-significant (**Figure 3C**).

### FoxP3 expression levels positively correlated with HO-1 levels in HIV-1 infected individuals

**Figure 4:**
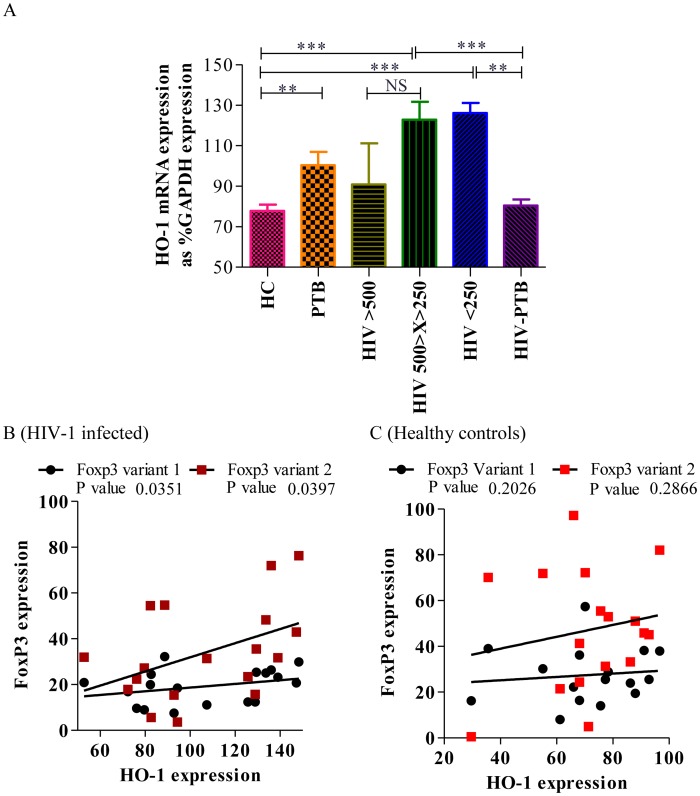
High HO-1 expression in the later stage of HIV-1 infection favors TB infection. **A.** The relative expression of the HO-1 gene was normalized using GAPDH levels in all study groups. All data are expressed as mean±SEM values. Significant differences are represented as the p value <0.05 (*). **B.** Represents scatter plot depicting significant positive correlation between HO-1 and FoxP3 gene expression in HIV-1 infected individuals. **C.** Scatter plot showing no significant correlation between HO-1 and FoxP3 variant 1 & 2 expression in PBMCs of healthy controls. The solid lines represent linear regression of points. Rho and p values are from the Spearman rank correlation test.

Since HO-1 has been implicated in the activation as well as induction and/or expansion of Tregs with its constitutive expression in human peripheral blood Tregs but not in resting CD4^+^CD25^−^ non-Tregs, we have also studied the relationship of HO-1 and FoxP3 splice variant levels in our study groups. Though, there was a significantly higher expression of HO-1 in HIV-1 infected individuals as compared to healthy controls, but there was no significant change in HO-1 expression in HIV-1 infected individuals with disease progression (**Figure 4A**). Whereas, HIV-PTB co-infected individuals had significantly lower (mean±SEM: 80.49±2.9) expression of HO-1 in comparison to HIV-1 infected individuals with similar CD4 count (<250 cells/µl (p = 0.0022)). Spearman's correlation analysis revealed a significant positive correlation between HO-1 and both FoxP3 splice variants in HIV-1 infected individuals only [variant-1: r = 0.4365, p = 0.0351, variant-2: r = 0.4241, p = 0.0397 (**Figure 4B**)] but not in healthy controls [variant 1: r = 0.2235, p = 0.2026, variant 2: r = 0.4171, p = 0.2866 (**Figure 4C**)]. These findings indicate a possible role of HO-1 in FoxP3 mediated suppression of T-cell activation in HIV-1 infected individuals.

### Lower HO-1 levels in HIV-PTB co-infected individuals correlate significantly with higher expression of NF-κB and HIV co-receptors CCR5 and CxCR4 on Tregs

**Figure 5:**
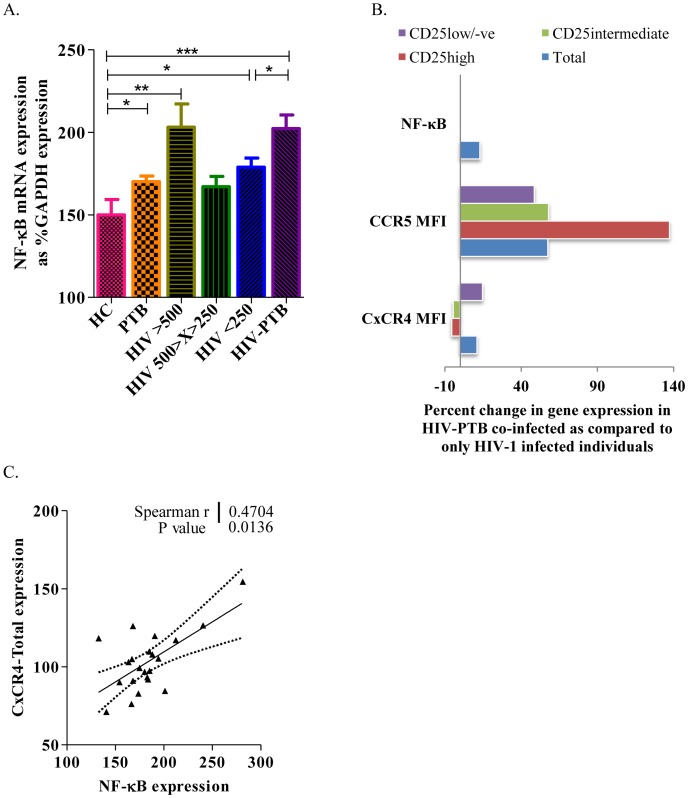
Higher NF-κB expression in HIV-PTB co-infected individuals modulate HIV-1 co-receptor expression on CD4 T-cell sub-populations. A. Represents bar diagram depicting relative expression of NF-κB gene mRNA (normalized using GAPDH levels) in all studied groups. Data were expressed as Mean ± SEM and p-value < 0.05 (*) was considered significant. B. Represents bar diagram depicting relative change in the expression of NF-κB gene mRNA (normalized using GAPDH levels), CCR5 and CxCR4 expression on CD4 T-cell sub-populations in HIV-PTB co-infected individuals as compared to only HIV-1 infected individuals with similar CD4 count. C. Scatter plots depicting the correlation of NF-κB with CxCR4 mRNA expression in PBMCs of HIV-1 infected individuals. The solid line represents the linear regression of points and dotted lines represents 95% confidence band for their mean values. Rho and p values are from the Spearman rank correlation test.

HO-1 induction provides important protection from oxidative stress and cellular damage. Lower levels of HO-1 in PTB co-infected individuals can up-regulate redox sensitive NF-κB, which is known to have a binding site on the Long Terminal Repeats [11] of the integrated HIV-1 provirus and induces its replication. We found significantly higher levels of NF-κB expression in HIV-PTB co-infected individuals (mean±SEM: 202.3±8.3, p = 0.0152) when compared to HIV-1 infected individuals with comparable CD4 counts [<250 cells/µL; mean±SEM: 178.9±5.5 (**Figure 5A**)].

NF-κB is known to promote expression of HIV-1 co-receptors, CCR5 and CxCR4, via binding to the target DNA elements and positively regulating the transcription of these genes. Our results are in concordance with the above observation, showing higher expression of NF-κB in HIV-PTB co-infected individuals (up by 13% as compared to only HIV-1 infected individuals with CD4 count <250 cells/µL, p = 0.0152) correlating with 57% higher expression of CCR5 on CD4 T-cells in these subjects (**Figure 5B**). This increase in the CCR5 expression was observed among all Foxp3^+^ Tregs, both CD25^+^ as well as CD25 negative cells, although the expression was more prominent in CD25^high^ cells.

Similarly, expression of CxCR4 (another coreceptor for HIV-1) was also increased in HIV-PTB co-infected individuals by 15%. This increase was seen only in CD25^low/negative^ cells but not in CD25^high^ or CD25^intermediate^ cells (**Figure 5B**), although the mRNA levels of CxCR4 correlated positively with NF-κB in HIV-1 infected individuals [r = 0.4704, p = 0.0136 (**Figure 5C**)].

### IL-6 levels negatively correlate with CCR5^+^ Treg frequency in therapy naïve HIV-1 subjects

**Figure 6:**
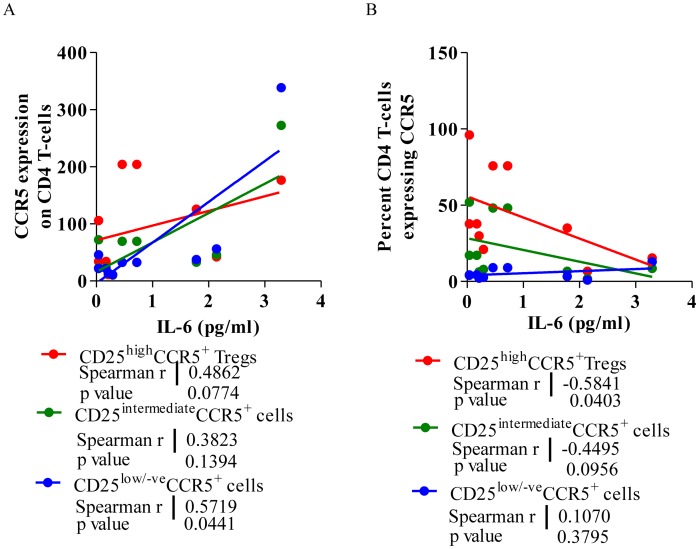
IL-6 levels bear a significant negative correlation with higher CCR5 expressing Tregs. **A.** A representative scatter diagram depicting the Spearman correlation between TNF-α (pg/ml), CCR5 expression (MFI) on CD4+CD25^high^ Tregs, CD25^intermediate^ and CD25^low/negative^ effector cell in PBMCs from HIV-1 infected individuals. **B.** A representative scatter diagram depicting the Spearman correlation between IL-6 (pg/ml), percent CCR5+ cell on CD4+CD25^high^ Tregs, CD25^intermediate^ and CD25^low/negative^ effector cell in PBMCs from HIV-1 infected individuals. The solid line represents the linear regression of points. Rho and p-values are from the Spearman rank correlation test.

Interleukin-6 is known to initiate the activation events including phosphorylation of JAK kinases resulting in translocation of NF-κB to the nucleus. With previous observation of NF-κB modulating HIV-1 co-receptors expression, we investigated the role of IL-6 in modulating their expression on CD4 T-cell populations in HIV-1 individuals. Spearmen's correlation analysis revealed a significant positive correlation between CCR5 expression on CD4 T-cells and levels of IL-6 produced on activation (r = 0.5352, p = 0.05). This correlation was also evident with respect to CCR5 expression on CD25^high^ (r = 0.4862, p = 0.0771) as well as CD25^low/negative^ cells (r = 0.5719, p = 0.0421, **Figure 6A**). Interestingly, we found a negative correlation between frequency of CCR5^+^ CD25^high^ Tregs and level of IL-6 (r = −0.5841, p = 0.0381, **Figure 6B**), indicating a specific depletion of CD25^high^ cells probably due to enhanced infectivity and faster depletion of these cells expressing higher number of CCR5 receptors in presence of higher levels of IL-6.

There was however no correlation between IL-6 levels and CxCR4 expression on the CD4 T-cells (p = 0.1217), Tregs (p = 0.0996), CD25^+^ cells (p = 0.0631) or CD25^low/negative^ cells (p = 0.1926) in HIV-1 subjects.

### TNF-α level positively correlate with CCR5 and CxCR4 expression on Tregs in therapy naïve HIV-1 subjects

**Figure 7:**
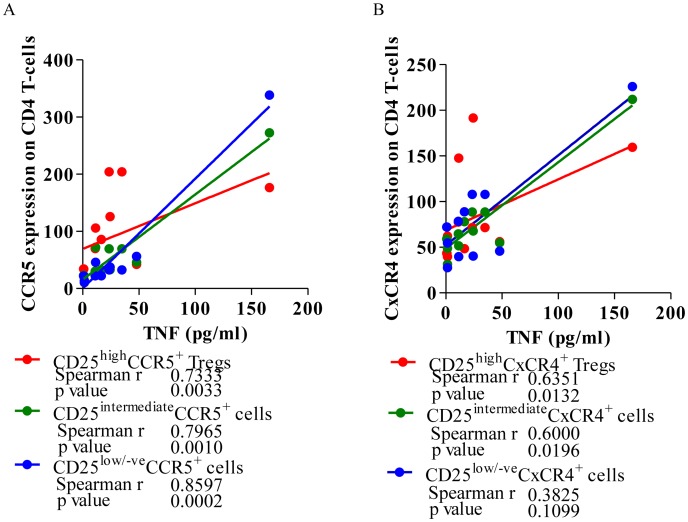
TNF-α level bear a significant correlation with CCR5 and CxCR4 expression on CD4 T-cells. **A.** A representative scatter diagram depicting the Spearman correlation between TNF-α (pg/ml), CCR5 expression (MFI) on CD4+CD25^high^ Tregs, CD25^intermediate^ and CD25^low/negative^ effector cell in PBMCs from HIV-1 infected individuals. **B.** A representative scatter diagram depicting the Spearman correlation between TNF-α (pg/ml), CxCR4 expression (MFI) on CD4+CD25^high^ Tregs, CD25^intermediate^ and CD25^low/negative^ effector cell in PBMCs from HIV-1 infected individuals. The solid line represents linear regression of points. Rho and p-values are from the Spearman rank correlation test.

There was highly significant positive correlation between levels of TNF-α production on activation with T-cell mitogen and CCR5 expression on CD4 T-cells [r = 0.8597, p = 0.0002 (**Figure 7A**)], suggesting possible involvement of TNF-α in controlling the CCR5 expression on CD4 T-cells, probably because the CCR5 promoter itself has an NF-κB binding site [12], which could potentially respond to TNF-α. This was similar when CD4 T-cells were further divided into Tregs and effector cells based on CD25 expression.

Overall there was however no such relationship observed between TNF-α levels (pg/ml) and CxCR4 expression on CD4 T-cells in HIV-1 individuals [r = 0.4526, p = 0.0698 (**Figure 7B**)]. But when looking into the sub-populations of CD4 T-cells, we found significant positive correlation of TNF-α levels with CxCR4 expression on CD25^high^ Tregs (r = 6351, p = 0.0132) and CD25^+^ activated T-cells (r = 0.6, p = 0.0196). These findings are further supported by significant positive correlation between NF-κB and CxCR4 mRNA expression in PBMCs of HIV-1 infected individuals (ref to Figure 5C).

### High TNF-α levels associate with up-regulated apoptosis of lymphocytes in HIV-1 infected individuals

**Figure 8:**
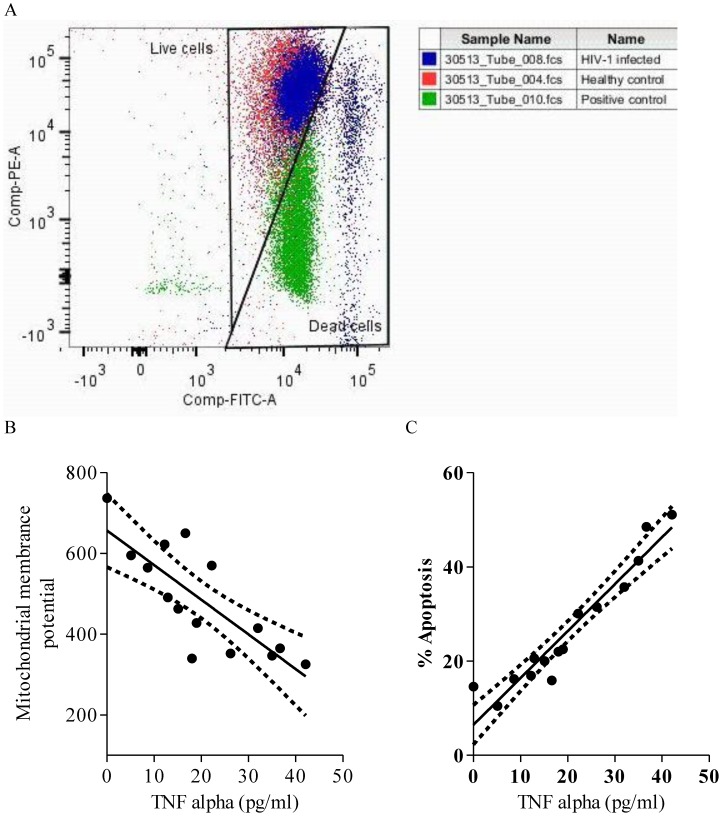
Higher plasma TNF-α levels up-regulate apoptosis of peripheral lymphocytes in HIV-1 infected individuals. **A.** Representative overlay dot plot showing JC-1 staining of peripheral blood lymphocytes depicting a shift of fluorescence emission from red (FL2) to green (FL1) indicating loss of mitochondrial membrane potential, which is associated with apoptosis in HIV-1 infected individuals (blue). Negative control (red, healthy control) and positive control (green, protonophore FCCP treated) were used to set the gate for dead cells. **B.** Representative scatter plots depicting significant negative correlation between TNF-α expression and mitochondrial membrane potential (ΔΨ_m_) in HIV-1 infected individuals. **C.** Representative scatter plots depicting significant positive correlation between TNF-α expression and cells undergoing apoptosis in HIV-1 infected individuals. Solid line represents linear regression of points and dotted lines represents 95% confidence band for their mean values. Rho and p-values are from the Spearman rank correlation test.

As TNF-α showed positive correlation with HIV-1 co-receptors, CxCR4 and CCR5, via NF-κB pathway, its expression could modulate the course of HIV-1 disease progression. We investigated how TNF-α influences the decline of CD4 count, one of the prime cells affected in the lymphocytes in HIV-1 infected individuals in terms of decrease in mitochondrial membrane potential (ΔΨ_m_), which is an indicator of apoptotic cells (**Figure 8A**). We found a highly significant negative correlation between ΔΨ_m_ and levels of TNF-α (pg/ml) in HIV-1 infected individuals (r = 0.7821, p = 0.0003) indicating its role in apoptosis (**Figure 8B**). This was supported by a significant positive correlation between TNF-α and the number of -cells undergoing apoptosis (r = 0.9571, p = 0.0001 (**Figure 8C**).

## Discussion

*Mtb* continues to be one of the most dreaded pathogens around the world and its cohabitation with HIV exacerbates its disease progression. However, our current knowledge about the mechanisms of interaction of the two pathogens still has many gaps that need to be bridged in order to develop preventive measures against the two diseases. Much can be learnt about the relationship between HIV and *Mtb* co-infection by monitoring the immune profile of the host during co-infection and conditions facilitating HIV-1 replication. As CD4^+^ T-regulatory cells are known to modulate the activity of CD4 T-cells, we have carried out investigations to unravel the possible role of Tregs in HIV-1 infected individuals at different stages of the disease along with its effect on the synergistic relationship of the two pathogens. In order to understand the role of Tregs in HIV-PTB co-infected individuals, we have studied the inter-relationship of these suppressor cells with various factors such as HO-1, NF-κB and HIV-1 co-receptors, highlighting how the virus manipulates the host machinery during *Mtb* co-infection to its advantage.

The role and frequency of Tregs in HIV-1 infected individuals have long been controversial. Some studies suggest that Tregs get depleted by HIV infection [13], [14], [15], while others indicate that the proportion of these cells may be rather increased [9], [16]. This difference in opinion could be due to differences in the stage of the disease at the time of sampling by the researchers or infection with different HIV-1 strains. In the present study, we have analyzed the frequency and suppressive function of Tregs in ART/ATT naive HIV-1 subtype-C infected subjects at different stages of the disease and shown how the presence of Mtb could affect these cell populations. There was a significant decrease in the frequency of Tregs in the early phase of the HIV-1 infection correlating with significant decrease in Foxp3 expression. These findings indicate that in the initial stage of HIV infection, there is a preferential killing/decrease of Foxp3 expressing CD4 T cells in the peripheral blood. The decreased FoxP3 expression may contribute to higher levels of immune activation thereby facilitating HIV-1 replication that is seen in the early phase of the disease. However, with HIV-1 disease progression, there was an up-regulation of both, the frequency of these Tregs as well as the level of FoxP3 expression. We noticed a significant inverse relationship of CD4 count with respect to the frequency of Tregs and FoxP3 expression, making these subjects more vulnerable to active tuberculosis in this immune-compromised state. These findings correlate and are in agreement with the immune-suppressive nature of Tregs. There was a significant negative correlation between percent Treg frequency and T-cell proliferation, exhibiting intact suppressive activity inherent in these cells. Although, the hallmark of HIV infection is gradual loss of CD4+ cells that results in immunodeficiency state, which further seem to get compounded by the down regulation of CD4 T cell activity with increase in Treg number, making the infected individual severely immune-compromised thereby increasing its susceptibility to opportunistic infections like *Mtb*.

With the onset of PTB co-infection in the dampened immune environment of HIV-1 infected individuals, we observed further significant increase in Foxp3 expression in CD4 T-cells of co-infected individuals in comparison to individuals infected only with HIV-1 but similar CD4 count (<250 cells/µl). Though statistically non-significant, there was also an increase in the Treg frequency in the PTB co-infected individuals when compared with only HIV-1 infected individuals with compatible CD4 cell count, indicating a possible role of *Mtb* in inducing Foxp3 expression as well as expansion of Tregs. Quantitatively up-regulated Foxp3 expression per cell (MFI) in Tregs and significantly increased frequency of FoxP3 positive cells among CD4 T-cells of PTB and HIV-PTB co-infected individuals compared to healthy controls, confirmed this observation.

Realizing the controversy in literature regarding the quantitative expression of FoxP3 mRNA in HIV-1 infected individuals [3], [13], [17], we also found no significant difference in relative FoxP3 mRNA expression within the HIV-1 groups at different levels of disease progression, although there was a visible decrease in FoxP3 mRNA expression in PTB subjects in comparison to healthy controls, though statistically non-significant. These results were however in contrast to protein levels assessed by flowcytometry. Absence of correlation between FoxP3 protein and total mRNA levels raised a question and prompted us to look further into the splice variants for a possible answer, as the humans express two main isoforms of FoxP3, variant-1 (NM_104004) and variant-2 (NM_001114377), either of which can confer regulatory function when strongly over expressed [18], [19], [20]. The main deletional isoform of FoxP3, variant-2 however, lacks the proline-rich exon 2, which encodes the Leu-X-X-Leu-Leu motif that is required for the binding to the transcription factor retinoic acid receptor-related orphan receptor-α (RORα) [21], and lacks amino-terminal residues that may mediate the interaction with nuclear factor of activated T cells (NFAT) resulting in transcriptional repression [11], [22]. The role of FoxP3 variant-2 in human Treg biology remains unclear. Interestingly it was FoxP3 variant-1 mRNA expression, which was significantly higher in PTB individuals in comparison to healthy controls. Further, its expression was also significantly higher in the HIV-PTB co-infected individuals when compared to HIV-1 individuals in advanced stage of disease (CD4 count <250 cells/µL). These results were in agreement with the flowcytometry results. On the contrary, there was no significant difference in the expression of FoxP3 variant-2 between the studied groups. These results indicate a direct relationship between FoxP3 mRNA expression and FoxP3 protein expression (MFI) for all the studied groups only with variant-1 and not with variant-2 or whole FoxP3 gene expression.

The FoxP3 expression and Treg function have been linked with hemoxygenase-1 (HO-1), which is implicated in the activation as well as induction and/or expansion of Tregs, as it is constitutively expressed in human peripheral blood Tregs but not in resting CD4^+^CD25^−^ non-Tregs [23], [24], [25], [26], [27], [28], [29]. Since HO-1 derived carbon monoxide can induce the DosR dormancy regulon in mycobacteria, leading to latency and survival of this organism inside the host granuloma, we hypothesized that such a situation could contribute to 20–30 times higher lifetime risk of developing active tuberculosis in HIV infected individuals. Hence, we studied the HO-1 mRNA expression in patient groups and found a significant increase in the expression of HO-1 in HIV patients. These results are in line with previous findings where HO-1 has been shown to be induced by oxidative stress, and pro-inflammatory cytokines that are characteristic of HIV-1 infection. Thus, the immune-compromised state was further exacerbated by a significant increase in HO-1 expression (via carbon monoxide) in HIV-1 (but PTB negative) group irrespective of the stage of the disease, where HO-1 is also known to play a role in promoting latency and survival of Mycobacteria inside the host granuloma. The increase in HO-1 makes the environment favorable for mycobacterial infection. On the other hand, in HIV-PTB co-infected individuals, we observed significantly lower HO-1 expression when compared to only HIV-1 infected subjects with similar CD4 count (<250 cells/µL). It is evident from this observation and previous findings that low HO-1 levels would lead to decreased levels of DosR gene expression in mycobacterium in co-infected subjects and could be a possible reason why these subjects have active tuberculosis. But what triggers the lowering of HO-1 gene expression in these subjects still needs to be further investigated. Is it the lowering of HO-1 expression that causes active tuberculosis or active tuberculosis leads to lowering of HO-1 is still a question that needs further investigation. Such a decrease in HO-1 expression by *Mtb* in HIV co-infected subjects results in the increase in redox stress, which has been shown to reactivate latent HIV-1 provirus [30] resulting in high plasma viral load.

The above findings further prompted us to look at the redox sensitive NF-κB expression [31], [32], which on activation translocate into the nucleus [33], where it binds to the Long Terminal Repeats [11] of the integrated HIV-1 provirus and induces its replication [34], [35], [36]. Interestingly, we observed that the presence of *Mtb* infection in HIV-1 individuals significantly increased NF-κB expression, thereby making the conditions favorable for HIV-1 replication. This is further supported by the fact that corresponding to a significant decrease in HO-1 in HIV-PTB individuals, there was a significant increase in NF-κB in comparison to HIV-1 infected individuals with similar CD4 count (<250 cells/µl). These data suggest that a decrease in HO-1 activity during *Mtb* co-infection in HIV-1 individuals can reactivate latent HIV-1 which may contribute to high viral load as well as faster disease progression towards AIDS.

Inflammatory cytokines like TNF-α and IL-6, that are known to activate and induce NF-κB expression, can also modulate the co-receptors of HIV. Interestingly, TNF-α showed highly significant positive correlation with CCR5 expression on CD4 T-cells in ART naive HIV-1 infected individuals, where as IL-6, showed moderate association with CCR5 expression on the CD4 T-cells. On the other hand, the TNF-α showed moderate association with CxCR4 expression on Tregs and CD25^+^ activated CD4 T-cells. Moreover, the NF-κB can also bind to CxCR4 promoter and activate its transcription [37], which was also substantiated by significant positive correlation between NF-κB and CxCR4 mRNA expression in PBMCs of HIV-1 infected individuals.

As TNF-α receptor/ligand pairs initiate a variety of biological responses, primarily through activation of inducible transcription factors such as NF-κB which in turn can affect the expression of HIV-1 co-receptor on the cell surface. We found a significant increase in the expression of NF-κB in HIV-PTB co-infected individuals when compared to HIV-1 infected individuals with comparable CD4 count i.e. <250 cell/µl. This could be one of the mechanisms how *Mtb* manipulates the host machinery towards rapid HIV-1 replication. Individuals carrying high TNF-α producer haplotype (CAG) have earlier been shown to be associated with faster disease progression due to increased apoptosis of T-cells in HIV-1 infected fast progressors (personal unpublished observation), we also found here that high plasma TNF-α levels were associated with increased apoptosis in lymphocytes.

To summarize, the present study has provided the experimental basis which highlights the interaction of various genes during HIV and *Mtb* co-infection in human host. This study, to the best of our knowledge, represents the first report reflecting the delicate link between NF-κB, TNF-α, IL-6, FoxP3 splice variants and HO-1 genes and their relationship with HIV disease progression and explains how *Mtb* co-infection modulates these links to the advantage of HIV. Apparently, HIV-1 makes conditions favorable for *Mtb* to grow in a co-infected host via increasing Treg numbers, FoxP3 expression and HO-1 expression. On the other hand, *Mtb* also facilitates HIV-1 replication by increasing redox stress (thereby reducing HO-1), increasing expression of co-receptors on CD4 T-cells *via* IL-6, TNF-α and NF-κB pathway leading to increased HIV-1 replication and enhanced HIV disease progression.

A close synergistic relationship between HIV-1 and *Mtb* mediated by interactions between few immune-modulatory genes as depicted in this study, highlights how these gene interactions play an important role in making the environment conducible for each other. These interactions need further attention and may suggest therapeutic intervention to prevent *Mtb* co-infection and faster HIV-1 progression.
